# Growth Concerns in Coffin–Lowry Syndrome: A Case Report and Literature Review

**DOI:** 10.3389/fped.2018.00430

**Published:** 2019-01-25

**Authors:** Ying Lv, Liuyan Zhu, Jing Zheng, Dingwen Wu, Jie Shao

**Affiliations:** ^1^Department of Pediatric Health Care, The Children's Hospital, Zhejiang University School of Medicine, Hangzhou, China; ^2^Department of Gene Screening Laboratory, The Children's Hospital, Zhejiang University School of Medicine, Hangzhou, China

**Keywords:** RSK2, Coffin–Lowry, growth retardation, pervasive development disorder, growth hormone

## Abstract

Mutation of RPS6KA3 can induce Coffin–Lowry syndrome, an X-linked syndrome. The case here reported manifests its signature characteristic of short stature, facial dysmorphism, development retardation, hearing defect. The mutation of RPS6KA3 we detected by NGS analysis is c.2185 C > T. The short stature is a noteworthy problem we discuss here to improve the patient's growth and development. The efficacy and safety of application of growth hormone analogs on patients with CLS are not confirmed and need to be carefully considered.

## Background

A growing number of studies have demonstrated that RPS6KA3 is a molecular etiology of Coffin–Lowry syndrome (CLS) (1, 2), an X-linked semidominant syndrome which was first reported by Coffin in 1966 and characterized by short stature, facial dysmorphism, severe-to-profound intellectual disability (ID), motor developmental delay, progressive skeletal deformities, dental disorders, hearing defect and other congenital deformity in males, with intellect ranges from normal to severely impaired in heterozygous females (3–6).

The protein responsible for RPS6KA3 is the ribosomal protein S6 kinase polypeptide 3 (RSK2), which is a serine/threonine kinase of a family of mitogen-activated protein kinases (4). The RSK2 protein is composed of two functional kinase domains that are activated in a sequential manner by a series of phosphorylations, such as PDK docking site (4). RSK2 has been found to be related with prompting cell proliferation, blocking cell differentiation and protecting cells from apoptosis (7, 8). To date, there have been reports of cases with different symptoms and with over 128 distinct mutations in the RSK2 gene split into 22 exons of chrXp22.2 (RPS6KA3) (4, 5). Recently, the mutations of RPS6KA3 have been found to be related with heart disease, osteosarcoma, foramen magnum compression, Drop episodes and so on (7, 9–12).

Worldwide, the speculated prevalence of CLS may be 1/50,000–1/100,000 and about 70–80% of probands have no family history while 20–30% have more than one affected family member as reported (13). As yet, there is no cure for this pathology. Here, we report a mutation of RPS6KA3 at Xp22 in a Chinese boy. The phenotypic appearance was typical, including growth and development retardation, as was described in the previous reports.

## Case Presentation

**Figure 1:**
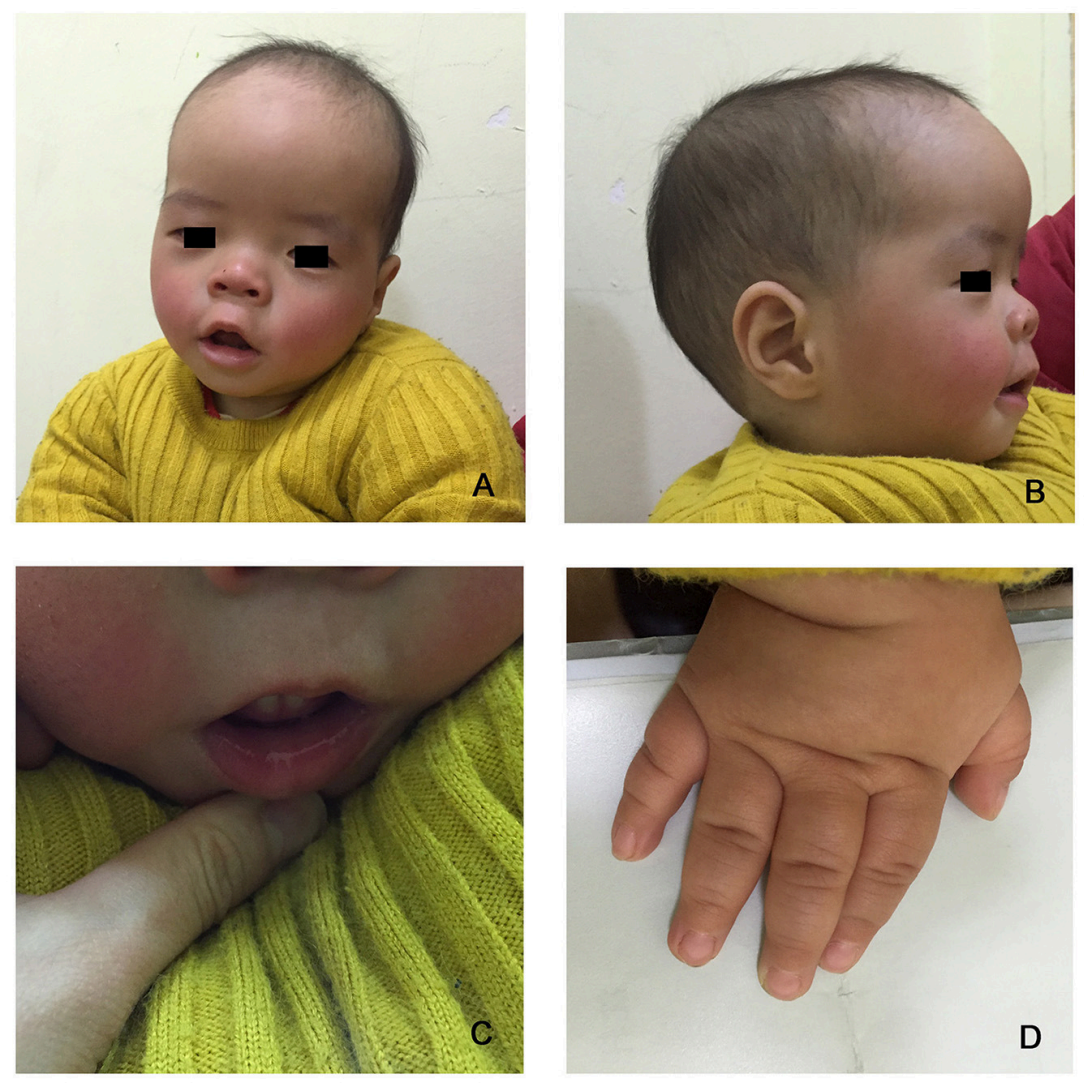
The distinctive facial dysmorphisms of a Coffin–Lowry syndrome boy. A side view of the patient's face **(B)**, prominent ears, widely spaced eyes, down slanted palpebral fissures, short nose with broad columella, thick alae nasi and septum, thick and everted underlip **(A,C)**. **(D)** The hands are short and with puffy tapered finger, small terminal phalanges, and nails.

**Figure 2:**
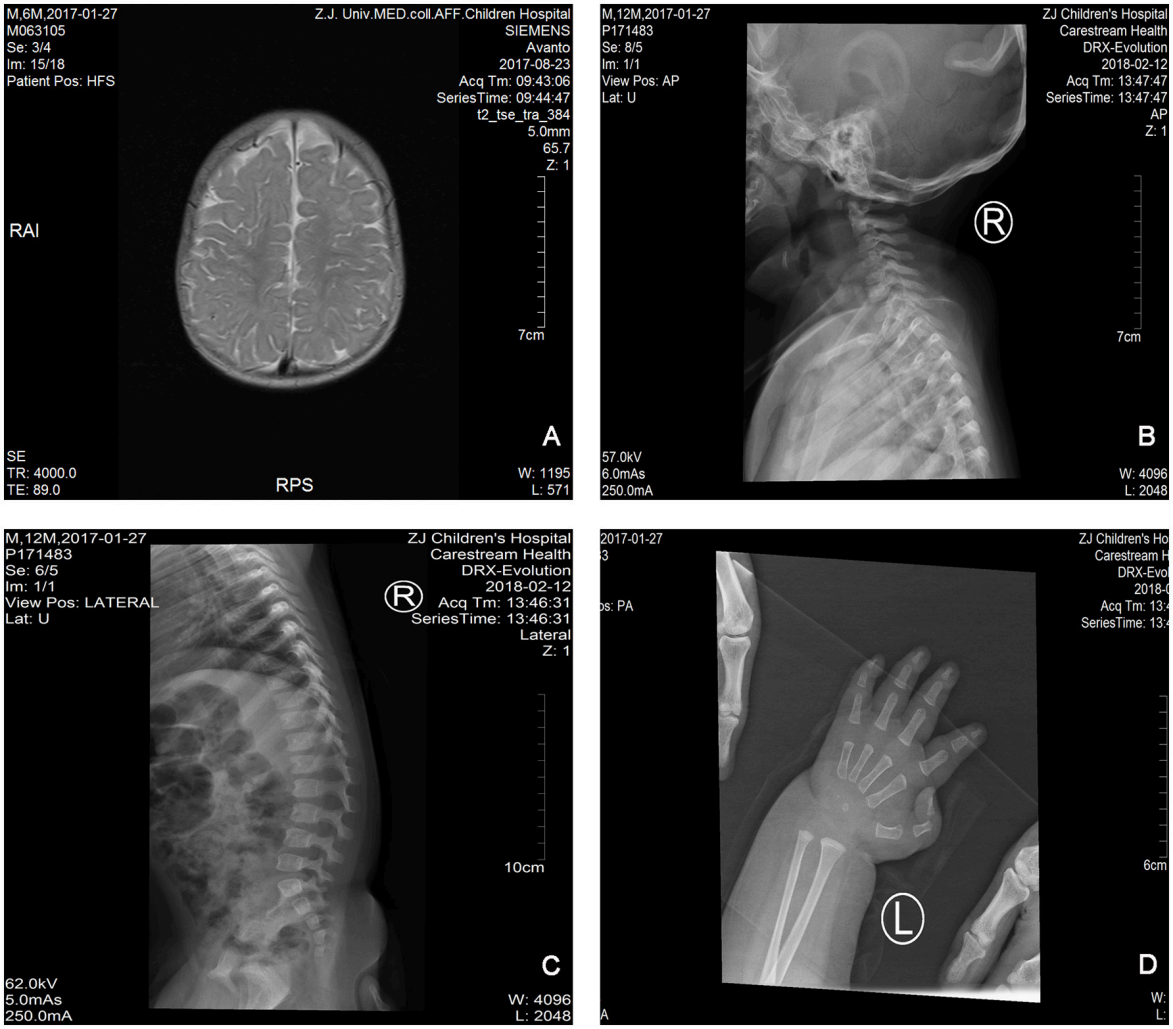
The magnetic resonance imaging (MRI) and X-ray images of the Coffin–Lowry syndrome case. **(A)** The MRI of cerebral development; **(B,C)** Cervical and spinal canal X-ray photographs. **(D)** Nodular hyperplasia of finger tail end.

The proband is a 12-months-old boy with typical facial dysmorphism, hearing defect and bony abnormality (Table 1). He was born after a normal pregnancy and delivered with birth weight of 2.9 kg (10th percentile) and birth length of 45 cm (3rd percentile) at 38 weeks, compared with the WHO Child Growth Standards in 2006. The facial appearance presents bulging forehead, prominent ears, widely spaced eyes, down slanted palpebral fissures, short nose with broad columella, thick alae nasi and septum, thick and everted underlip (Figures 1A,B). The deciduous teeth were erupted at 8 months old (not delayed) (Figure 1C). The hands are short, fleshy, and with remarkably hyperextensible fingers that taper from wide to narrow with small terminal phalanges and nails (Figures 1D, 2D). But there was no deformity of his foramen magnum or spine column (Figures 2B,C). The weight at 12 months is 8.2 kg and height is 68.2 cm (<-3.17 z score, WHO). The bone metabolism and IGF-1α is disturbance (Vit D 45.2 nmol/L, IGF-1α < 25 ng/mL). He started sitting alone at 9 months and couldn't stand unaided until 12 months of age. At 12 months of age, his intelligence quotient (IQ) was 56 according to the Gesell Developmental Schedules. He had difficulty remaining seated or concentrating during task completion. His auditory threshold of auditory brainstem response (ABR) is >85 db and is diagnosed as a hearing disorder. The magnetic resonance imaging (MRI) showed the dilation of bilateral ventricles and less cerebral white matter (Figure 2A).

**Table 1:** The typical phenotype of Coffin–Lowry syndrome of proband.

1. Typical facial dysmorphism: bulging forehead, prominent ears, widely spaced eyes, down slanted palpebral fissures, short nose, everted underlip
2. Hearing defect: >85 db (both ears)
3. Hyperextensible fingers that taper from wide to narrow with small terminal phalanges and nails
4. Short stature: 68.2 cm at 1 year old
5. Mental retardation: IQ was 56
6. Hypoevolutism in motor development
7. Bone metabolism disturbance: Vit D 45.2 nmol/L
8. IGF-1α < 25 ng/mL

For genetic analysis, blood samples were obtained from the individual. The mother had given informed consent for her children. This research was approved by the bioethics committee for human gene analysis at the Zhejiang University.

**Figure 3:**
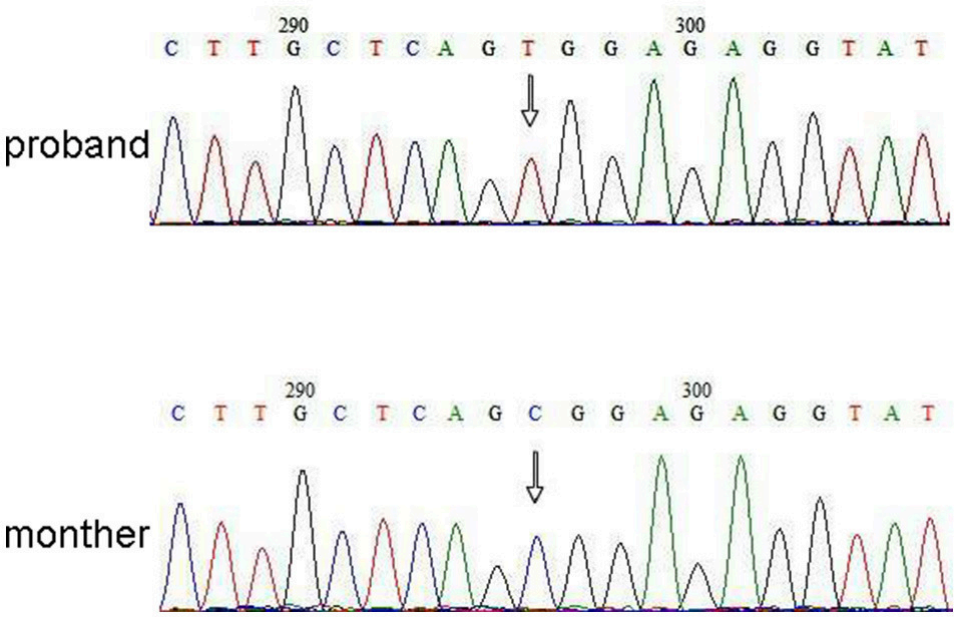
Sanger sequencing validation for the mutation of RPS6KA3 in the proband at chromosome Xp22. A mutation of c.2185 C > T was detected in imprinting control region from allele 290–300, which was not detected in proband's mother.

Next Generation Sequencing (NGS) analysis was performed using Agilent Human Genome panel (Agilent Technologies, Inc, Santa Clara, CA, USA). A c.2185 C > T at chrX20173554 was detected in the proband, which can cause p.Arg729Trp mutation and RSK2 instability (Figure 3). Subsequent analysis did not detect the same point mutation in other family members including mother, brother (Supplementary Material). This indicated that the micromutation is new but not inherited from the mother.

## Discussion

The case we present for genetics evaluation is a 12 months boy who was born after an uneventful pregnancy from healthy parents. However, his developmental age was delayed. Like other affected CLS patients, he had the typical phenotype observed in CLS including intellectual disability, retarded motor development, small stature, tapering fingers, hearing defect and characteristic facial features.

RPS6KA3 (OMIM 303600) is known to be mutated in patients with Coffin-Lowry syndrome. Exome c.2185 C > T variant at Xp22 was novel, segregated from the disease, and was predicted to be damaging using five different *in silico* software (SIFT, Polyphen, MutationTaster, FATHMM, LRT).

**Figure 4:**
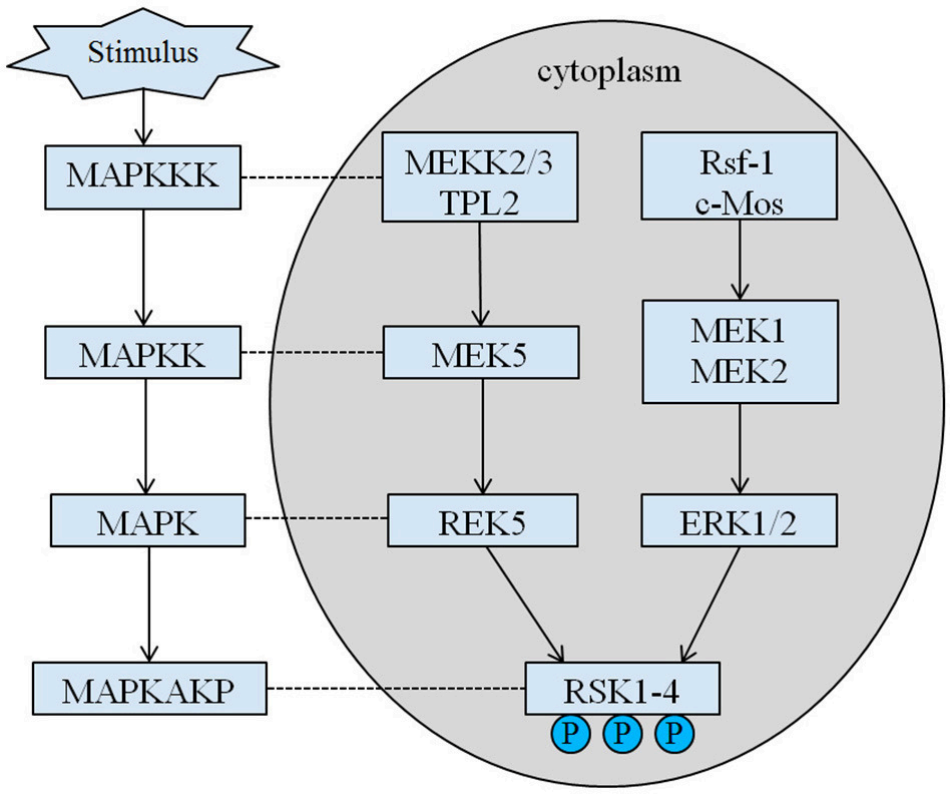
The proposed pathway of RSK related genes. Multiple pathological stimuli, such as intrauterine malnutrition, trigger downstream signaling cascades of MAPK, which modulate the cell proliferation and bone mineralization.

The inherited height of our proband is 167.5 cm because of his father's height is 173 cm while his mother's height is 150 cm (14). Whether the short stature of Coffin–Lowry syndrome could be treated with growth hormone analogs is still not clear. RSKs are serine/threonine kinases activated by ERK/MAPK and constitute four isoforms (RSK1–4) (15). RSKs act as downstream effectors of RTK/Ras/ERK signaling (Figure 4) (15–17). As the gene responsible for CLS, RSK2 play an important role in cell proliferation and migration. Full activation of RSK2 requires phosphorylation at multiple sites. Growth factors can stimulate ERK signal pathway to phosphorylated RSK2. As Ramos reported, ERK/RSK2 activating is involved in regulating cancer cell migrating (18).

What is more, RSK2 out of function is related with bone mineralization abnormal and also induces spine malformations and calcifications of ligamentum flavum, such as thoracic lordosis, scoliosis, kyphosis, and degenerative disc disease (19, 20). Previous reports identified the ERK/ RSK2 as a regulator of bone formation *in vivo* (7). Indeed, as Marques et al. reported, RSK2-deficient mice recapitulated the progressive bone loss due to decreased bone mineralization by the osteoblasts observed in patients with CLS (21). Although the differentiation of these cells *in vitro* was drastically blocked, the *in vivo* phenotype was clearly attributed to a cell autonomous decrease in the activity of the osteoblasts rather than a decrease in their numbers (7). Interestingly, RSK2 was recently shown to interact with TNF-RI to affected osteoblasts differentiation (18, 22). As we know, growth hormone can cause increased bone mineral density. The progress may aggravate calcifications of ligamentum flavum and skeletal deformity (23).

## Concluding Remarks

The present study is the first to report a Chinese case of CLS with mutation of RPS6KA3, as well as distinctly growth retardation, multiple facial abnormalities, intellectual and motor disabilities. We speculate that the application of growth hormone analogs may increase the incidence and invasiveness of cancers as well as the risk of spine malformation. If its efficacy and safety are not confirmed, the application of growth hormone analogs on patients of CLS should be especially cautiously considered. Long-term studies with relevant outcome will be essential in the future clinical research.

## Patient Consent

We obtained informed consent for publishing this case report and for using related images from patient's parents.

## Author Contributions

JS substantially contributed to the conception of this manuscript. YL, LZ, JZ, and DW contributed to the acquisition, analysis, or interpretation of data. YL drafted the manuscript. JS critically revised the manuscript for important intellectual content.

### Conflict of Interest Statement

The authors declare that the research was conducted in the absence of any commercial or financial relationships that could be construed as a potential conflict of interest. The reviewer BP and handling Editor declared their shared affiliation at the time of review.

## Supplementary Material

Figure S1 Sanger sequencing validation for the mutation of RPS6KA3 in the proband's brother.
